# Nerve Injuries in Craniofacial and Skull Base Surgery: Shared Mechanisms, Prevention, and Repair Strategies

**DOI:** 10.3390/brainsci16101062

**Published:** 2026-09-30

**Authors:** Ioannis Chatzistefanou, Sophia Tsokkou, Alexandros C. Liatsos, Panagiotis Tsolkas, Kyriaki Papadopoulou, Maria Florou, Martha Pyraki, Theodora Papamitsou

**Affiliations:** 1 University General Hospital “Attikon”, National and Kapodistrian University of Athens, 12462 Athens, Greece; ichatzistefanou@usa.com (I.C.);; 2 Department of Medicine, Faculty of Health Sciences, Aristotle University of Thessaloniki, 54124 Thessaloniki, Greece; 3 Department of Oral and Maxillofacial Surgery, Athens Naval Hospital, 11521 Athens, Greece; patsolkas@gmail.com; 4 Department of Neurosurgery, AHEPA University Hospital, Aristotle University of Thessaloniki, Kiriakidi 1, 54636 Thessaloniki, Greece; kiki_ppdpl@yahoo.com; 5 Second Department of Pediatric Surgery, “Papageorgiou” General Hospital, Aristotle University of Thessaloniki, 54636 Thessaloniki, Greece; 6 Laboratory of Histology-Embryology, Department of Medicine, Faculty of Health Sciences, Aristotle University of Thessaloniki, 54124 Thessaloniki, Greece

**Keywords:** cranial nerve injury, skull base surgery, craniofacial surgery, facial nerve, trigeminal nerve, intraoperative neuromonitoring, nerve repair, facial reanimation, inferior alveolar nerve

## Abstract

Background: Craniofacial and skull base procedures place cranial nerves at risk of injury, with consequences for movement, sensation, hearing, swallowing, vision, and quality of life. Methods: This narrative review integrates 46 original publications with six targeted references added during revision, addressing injury mechanisms, prevention, postoperative assessment, reconstruction, and rehabilitation across neurosurgery, otolaryngology, and maxillofacial surgery. Results: Recurring mechanisms include traction, compression, ischemia, thermal injury, direct trauma, and transection. Neuromonitoring contributes to localization, functional surveillance, and prognosis when paired with reproducible baseline recordings, appropriate modality selection, technical troubleshooting, and a predefined surgical response to alerts. Preoperative and postoperative electrophysiological testing complements but does not replace intraoperative monitoring. Recognized transection favors immediate tension-free reconstruction when feasible; an anatomically continuous nerve with an unfavorable electrical response requires contextual interpretation rather than automatic sacrifice. Rehabilitation combines target-organ protection, selective motor or sensory retraining, symptom control, and patient-reported assessment. Conclusions: The practical distinction is between a potentially reversible functional disturbance and confirmed structural discontinuity. Management should link monitoring changes to corrective action, preserve viable nerves, and coordinate timely reconstruction with rehabilitation. Heterogeneous, predominantly observational evidence does not support universal alert thresholds, a single reconstruction deadline, or guaranteed functional recovery.

## 1. Introduction

Craniofacial and skull base surgery encompasses a broad range of procedures for neoplastic, vascular, traumatic, congenital, and degenerative conditions involving some of the most anatomically complex regions of the human body. These operations require meticulous dissection within confined spaces that contain cranial nerves, sensory ganglia, brainstem pathways, and major vessels. Iatrogenic nerve injury therefore remains an important source of postoperative morbidity despite continued advances in microsurgery, endoscopy, imaging, and neurophysiological monitoring. The consequences extend beyond a focal neurological deficit and may include facial asymmetry, corneal exposure, oral incompetence, hearing loss, diplopia, dysphagia, dysphonia, altered taste, sensory loss, neuropathic pain, and marked deterioration in quality of life [1,2]. Recent systematic reviews spanning endoscopic skull base and broader head and neck procedures confirm that cranial nerve morbidity remains clinically important. In a recent review of 18,546 patients undergoing endoscopic endonasal skull-base surgery, 859 cranial nerve injuries were reported, corresponding to approximately 4.6 injuries per 100 patients [3]. The reported patterns of cranial nerve morbidity vary with approach, pathology, and the neural structures exposed [3,4,5].

Although the operative context differs across specialties, the underlying biology is shared. Mechanical traction, compression, ischemic compromise, thermal spread, direct instrument or bony trauma, and partial or complete transection are recurrent mechanisms in lateral and ventral skull base surgery, orthognathic procedures, mandibular resection, and facial reconstruction [1,2,6]. The resulting injury may range from transient conduction block to irreversible axonal and connective-tissue disruption. Functional outcome is influenced not only by the severity of the initial insult, but also by the anatomical segment involved, preservation of the distal motor end plate or sensory receptor field, time to recognition, and the availability of a tension-free reconstructive option. Experimental and clinical evidence from cerebellopontine angle surgery further emphasizes that structural preservation, microvascular integrity, and the regenerative environment jointly determine recovery [7].

Prevention and treatment are inherently interdisciplinary. Skull base surgeons must combine high-resolution anatomical planning with real-time interpretation of neurophysiological signals, whereas craniofacial and maxillofacial surgeons must account for the relationship between osteotomy lines, fixation, and peripheral trigeminal branches. When injury does occur, decisions concerning observation, decompression, direct neurorrhaphy, interposition grafting, donor nerve transfer, muscle transfer, and rehabilitation must be individualized. Contemporary reviews and guidelines increasingly emphasize that preservation, early recognition, reconstruction, and rehabilitation should be viewed as a continuous care pathway rather than separate phases [8,9,10].

Intraoperative neuromonitoring is an active component of this pathway, not merely a means of confirming nerve identity. Its preventive role depends on obtaining interpretable baseline signals, selecting modalities appropriate to the nerves at risk, recognizing deterioration during dissection, and responding before further manipulation compounds injury. Nevertheless, monitoring cannot guarantee preservation: current spread, stimulation distal to an injury, technical failure, and anesthetic effects can all alter interpretation. These limitations make coordination between the surgical, neurophysiological, and anesthesia teams essential [8,9,11,12].

The objective of this review is to connect shared injury mechanisms with procedure-specific prevention, interpretation of neurophysiological findings, and decisions about repair and rehabilitation. Its contribution is an interdisciplinary clinical synthesis rather than a new injury classification or a claim of comparative treatment efficacy. Particular attention is given to the distinction between anatomical continuity and physiological integrity, because this distinction determines whether the appropriate response is technical troubleshooting, modification of dissection, observation, or reconstruction. The intended contribution is therefore a decision-oriented reference for interdisciplinary practice and education, rather than a claim of originality in the underlying mechanisms or surgical techniques.

## 2. Materials and Methods

This narrative review evaluates the mechanisms, prevention, assessment, and reconstructive management of nerve injuries encountered in craniofacial and skull base surgery. The literature search was performed in PubMed/MEDLINE, Scopus, Embase, and Web of Science using combinations of the terms “skull base surgery”, “craniofacial surgery”, “facial nerve injury”, “trigeminal nerve injury”, “inferior alveolar nerve”, “lingual nerve”, “intraoperative neuromonitoring”, “nerve grafting”, “facial reanimation”, and “microsurgical nerve repair”. The reported search period in the databases was up to June 2026. Selection was restricted to English-language articles available in full text and was supplemented by examination of reference lists.

The scope of selection included publications addressing relevant nerve anatomy, mechanisms of operative injury, perioperative prevention or neurophysiological assessment, functional outcomes, reconstruction, or rehabilitation. Priority was given to systematic reviews, evidence-based guidelines, clinically relevant narrative reviews, and clinical cohorts with objective functional outcomes. Anatomical, imaging, technical, and preclinical reports were used for supporting context rather than as equivalent evidence of clinical treatment effectiveness. Non-English publications, reports without accessible full text, and publications without relevance to these domains were outside the stated scope. These scope-based criteria clarify the narrative selection approach; they do not represent prospectively registered systematic-review eligibility criteria. A total of 52 papers were used for the synthesis of this review.

**Figure 1:**
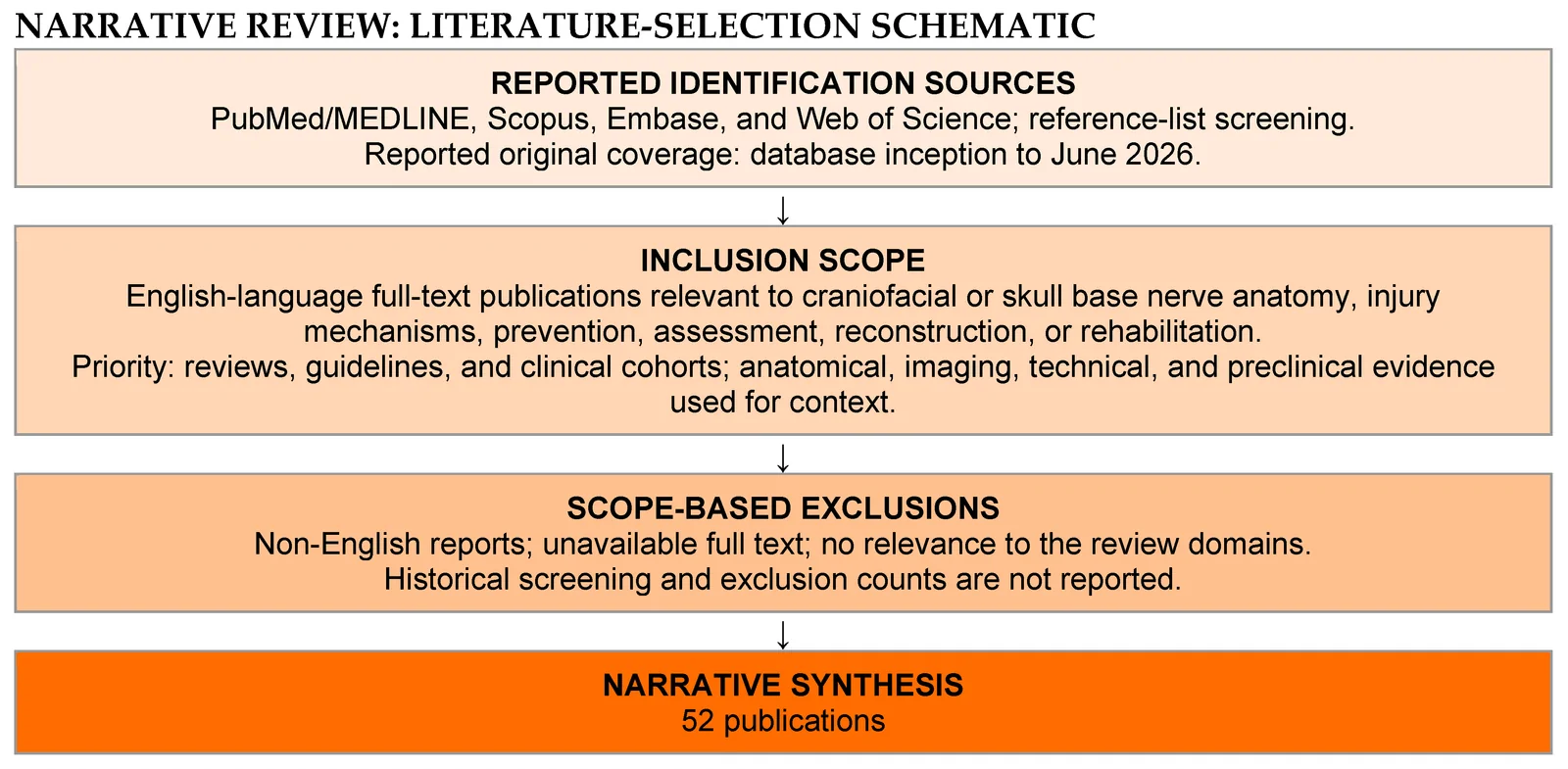
Reported sources and scope-based selection for the narrative review.

Figure 1 summarizes the reported sources, scope-based selection criteria, and composition of the revised narrative synthesis. The figure is a narrative selection schematic, not a PRISMA flow diagram, and the review does not claim exhaustive retrieval or systematic-review-level selection.

## 3. Surgical Neuroanatomy and Classification of Injury

The cranial nerves do not function as isolated cables. Anatomical communications between trigeminal, facial, vestibulocochlear, lower cranial, autonomic, and upper cervical nerves create variable extra-axial networks that may influence clinical presentation and surgical planning. The auriculotemporal-facial connection, communications between facial and trigeminal sensory branches, and the complex plexiform relationships among the glossopharyngeal, vagal, accessory, hypoglossal, and upper cervical nerves may partly explain unexpected patterns of preserved or impaired function after focal injury [13,14]. These variants are especially relevant during lateral skull base dissection, parapharyngeal and jugular foramen surgery, and donor nerve transfer.

From a biological perspective, injury severity is commonly described using the Seddon or Sunderland classifications. Neurapraxia represents a focal conduction block with preserved axonal continuity and is generally associated with the best potential for spontaneous recovery. Axonotmesis includes axonal disruption with variable preservation of the connective-tissue framework, whereas neurotmesis denotes complete disruption of the nerve and supporting sheaths. In practice, operative injuries often combine these mechanisms: a nerve may remain anatomically continuous yet contain areas of ischemia, intraneural scarring, fascicular disruption, or neuroma formation [1,2,4].

Anatomical continuity should therefore not be equated with normal function, and an absent intraoperative response should not automatically be interpreted as irreversible failure. Conversely, apparently minor manipulation may produce delayed dysfunction through edema, ischemia, or Wallerian degeneration. This distinction is central to intraoperative decision-making and to the timing of postoperative exploration [1,9,11,12].

## 4. Shared Mechanisms of Nerve Injury

**Figure 2:**
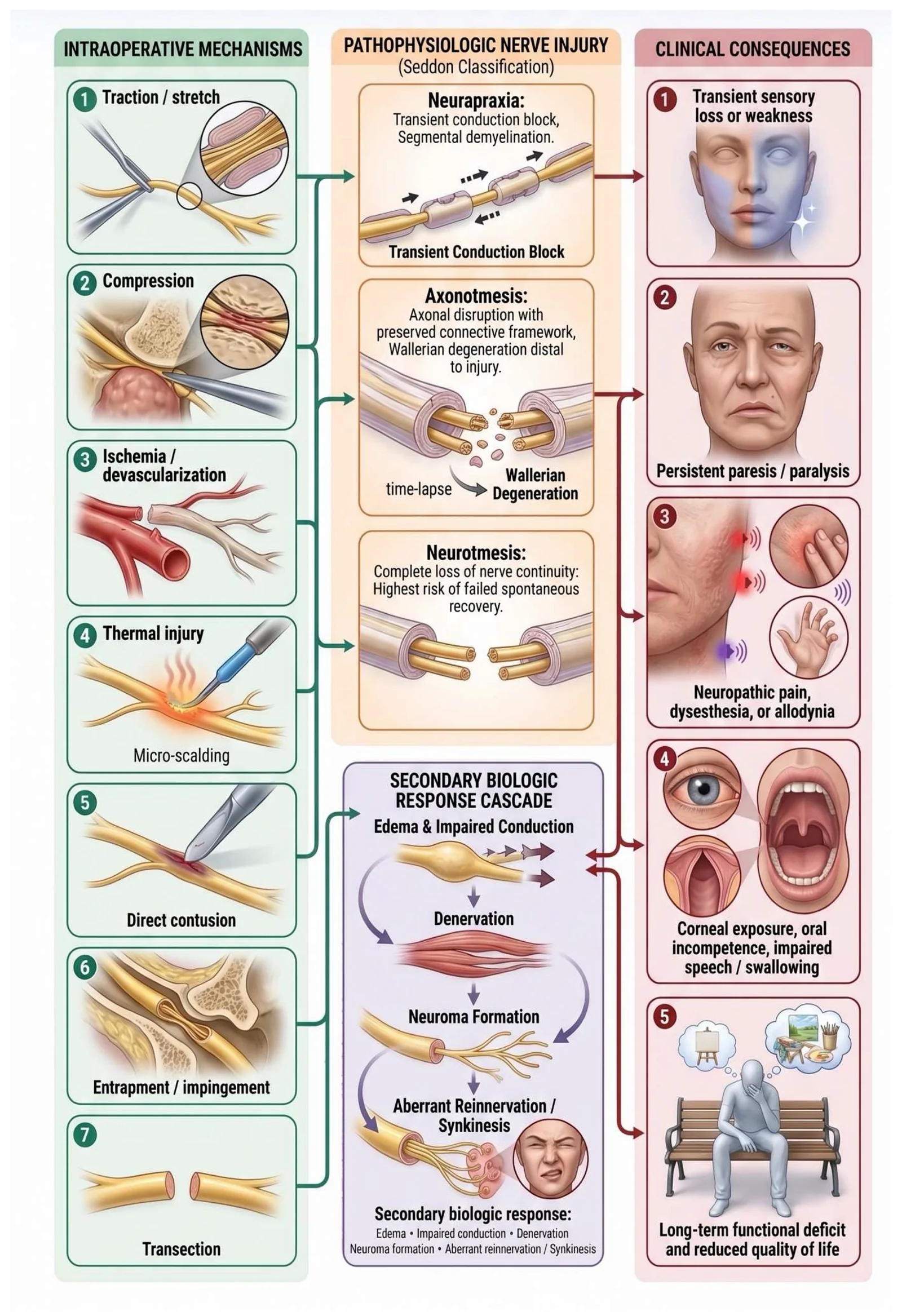
Shared mechanisms of nerve injury in craniofacial and skull base surgery.

The shared mechanisms, corresponding pathophysiological injury patterns, and principal clinical consequences are summarized in Figure 2.

### 4.1. Mechanical Traction and Compression

Stretch and compression are among the most frequent mechanisms of injury. During cerebellopontine angle tumor surgery, the facial nerve may be thinned, flattened, or displaced along the tumor capsule. Dissection can transfer traction to the transition between the relatively mobile cisternal segment and the fixed labyrinthine segment within the fallopian canal, creating a particularly vulnerable mechanical interface [1]. Large tumors, dense adherence, and loss of a clear arachnoid plane increase risk. Similar forces affect the cochlear nerve during mobilization of vestibular schwannomas and the abducens or oculomotor nerves during cavernous sinus and clival surgery. For large vestibular schwannomas, the tension between extent of resection and functional preservation is particularly pronounced, supporting individualized goals and explicit preoperative counseling [15].

In craniofacial surgery, peripheral trigeminal branches are injured by retraction, osteotomy, segment manipulation, fixation, or postoperative compression. The inferior alveolar nerve is particularly vulnerable during bilateral sagittal split osteotomy because it may remain attached to either bony segment, become compressed between segments, be stretched during advancement, or be injured by drills and screws. The lingual nerve is at risk during medial ramus dissection and bicortical fixation. The clinical spectrum ranges from transient hypoesthesia to persistent dysesthesia and neuropathic pain [16,17]. Orthognathic series and reviews additionally identify the magnitude and direction of skeletal movement, canal anatomy, nerve handling, and fixation strategy as modifiers of risk [18,19,20,21]. Pre-existing orthodontic compression or root-canal proximity may create an additional vulnerability that should be recognized before osteotomy [22].

### 4.2. Devascularization and Ischemia

Cranial nerve perfusion depends on small-caliber vessels that are easily compromised by coagulation, traction, skeletonization, or interruption of regional arterial branches. The facial nerve receives contributions from the anterior inferior cerebellar artery proximally and from petrosal and stylomastoid arterial systems distally. Coagulation near the nerve, excessive mobilization, and sacrifice of the vasa nervorum may cause dysfunction despite preserved gross continuity [1]. Hemostatic maneuvers can also inadvertently compromise neural perfusion. Acute compression for hemostasis can further reduce endoneurial blood flow and produce intraneural edema.

Ischemic injury is often difficult to recognize visually. Free-running electromyographic activity or a progressive change in evoked responses may provide an early warning, but signal interpretation must account for stimulation site, anesthetic factors, temperature, technical artifacts, and the possibility of distal stimulation below the level of injury [11,12]. Modern multichannel electromyographic datasets may support future development and validation of automated trend detection, but their clinical value depends on accurate annotation of surgical events and artifacts [23].

### 4.3. Thermal and Energy-Related Injury

High-speed drilling, ultrasonic instruments, and monopolar or bipolar electrosurgery can injure adjacent nerves through direct contact, transmitted heat, or vascular damage. Risk increases when the protective bony layer over a nerve is thin, eroded by tumor, or removed during decompression. Continuous irrigation, use of diamond rather than cutting burrs near critical neural structures, intermittent drilling, and avoidance of coagulation directly on or immediately adjacent to the nerve are basic preventive principles [1,6]. Cadaveric implant studies demonstrate that rotary instrument design and drilling behavior can influence the pattern and severity of inferior alveolar nerve trauma, while marked sphenoid sinus pneumatization may reduce the osseous protection of neural and vascular structures in ventral skull base corridors [24,25].

Thermal injury may be present as an abrupt loss of conduction without visible transection. Because no single universal monitoring threshold identifies all subclinical injury, operative teams must integrate signal changes with the exact surgical event and immediately pause, irrigate, release traction, optimize perfusion, and reassess stimulation parameters when a concerning change occurs [8,11,12].

### 4.4. Direct Contusion, Laceration, Transection, and Planned Sacrifice

Direct trauma may be produced by sharp instruments, drills, fixation screws, displaced bone, or an unfavorable fracture line. The most severe form is complete transection, whether inadvertent or required for oncological clearance. Planned sacrifice is encountered in facial nerve schwannoma, infiltrative temporal bone or parotid malignancy, and mandibular resection involving the inferior alveolar nerve. In these situations, the reconstructive strategy should ideally be planned before resection so that recipient and donor stumps are preserved, and reconstruction can be performed during the same operation [26,27]. In mandibular approaches, deliberate shielding of exposed mental nerve branches is a simple example of procedure-specific protection that can reduce direct instrument and retractor trauma [28].

When direct repair is possible, tension-free end-to-end neurorrhaphy is preferred. If a segment must be removed for oncological clearance, or if direct approximation would create tension, an interposition graft is required. Failure to recognize and address a transection at the index operation converts an immediately addressable reconstructive problem into a delayed injury complicated by fibrosis, retraction, and target-organ denervation [10,29].

The shared mechanisms of injury and their corresponding preventive responses are summarized in Table 1.

## 5. Procedure- and Nerve-Specific Injury Patterns

### 5.1. Lateral Skull Base and Cerebellopontine Angle Surgery

Facial and vestibulocochlear function dominate morbidity considerations in lateral skull base surgery. Vestibular schwannoma, cerebellopontine angle meningioma, epidermoid tumor, facial nerve schwannoma, paraganglioma, and temporal bone malignancy may distort, encase, infiltrate, or require sacrifice of the facial nerve. The operative objective has therefore evolved from anatomical preservation alone toward functional preservation, with real-time identification and prognostication becoming integral to modern surgery [8,9,11]. Consensus work on large vestibular schwannomas similarly stresses balancing tumor control with facial and cochlear nerve function rather than treating gross-total resection as an isolated endpoint [7,15].

An intact facial nerve may nevertheless be nonfunctional because of traction, ischemia, thermal injury, or severe intraneural damage. Conversely, weak or absent responses at the end of surgery do not invariably predict permanent paralysis. Evidence-based guidance consequently recommends routine facial nerve monitoring during vestibular schwannoma surgery, while cautioning against using a poor intraoperative electrical response as the sole indication for immediate reanimation when anatomical continuity is preserved [9].

### 5.2. Endoscopic Endonasal Skull Base Surgery

The endoscopic endonasal approach provides a direct route to the ventral skull base but places the optic apparatus, oculomotor nerves, trigeminal divisions, abducens nerve, vidian nerve, and lower cranial nerves at risk depending on the extent of exposure. Cranial nerve dysfunction may result from direct dissection, traction, thermal injury, vascular compromise, or postoperative cerebrospinal fluid pressure changes [6]. Risk reduction depends on detailed knowledge of foraminal anatomy, identification of the internal carotid artery, controlled drilling, and maintaining dissection within safe anatomical planes. A systematic review of endoscopic endonasal procedures confirms that the nerves at risk depend on the anatomical module and lesion extension, while delayed recognition of iatrogenic skull base injury can compound neurological morbidity [3,30]. Hyperpneumatized or dehiscent sphenoid variants warrant particular attention because they may narrow the margin for safe drilling around the optic canal, cavernous sinus, and carotid protuberance [25].

Triggered electromyographic mapping can assist localization of motor cranial nerves before direct visual identification, particularly in distorted anatomy. In a small comparative cohort of endoscopic endonasal tumor resections, triggered monitoring identified cranial nerves III, V, VI, VII, and XII and showed a directionally lower rate of postoperative deficit, although the difference was not statistically significant [31]. This evidence supports selective use in high-risk cases but also illustrates the need for larger prospective studies.

### 5.3. Orthognathic and Mandibular Surgery

In orthognathic surgery, neurosensory disturbance is most closely associated with the inferior alveolar nerve after sagittal split ramus osteotomy, while lingual and long buccal nerve injuries are less frequent but may be highly symptomatic. Standardized assessment is essential because reported incidence varies markedly according to whether outcomes are based on subjective patient report, objective sensory testing, or both [16,17,29]. The wider orthognathic literature also documents motor cranial nerve complications, rare multisystem cranial neuropathies after unfavorable Le Fort I fracture propagation, and injuries involving lingual, infraorbital, and optic pathways [18,19,20,21,32,33,34].

The clinical significance of trigeminal injury extends beyond numbness. Patients may experience allodynia, dysesthesia, tongue biting, altered taste, speech difficulty, impaired mastication, or distress related to an unpredictable sensory field [2,16,29,35,36]. Persistent neuropathic pain may be more disabling than complete anesthesia and should be explicitly assessed during follow-up. These symptoms may also be influenced by preoperative orthodontic nerve compromise and should not be reduced to a binary assessment of sensation present versus absent [22].

### 5.4. Dentoalveolar and Implant-Related Nerve Injury

Dental implant placement and third-molar surgery create additional risk to the inferior alveolar, lingual, mental, nasopalatine, and anterior superior alveolar nerves. Injury may result from direct drill or implant contact, compression within a confined canal, thermal osteotomy injury, or damage to variant anterior maxillary neurovascular channels. Systematic evidence supports careful three-dimensional assessment of the mandibular canal before implant placement, while anatomical variants of the nasopalatine canal and canalis sinuosus should be considered in the anterior maxilla [37,38].

For mandibular third molars, interruption of canal cortication, lingual canal position, and canal deformation on cross-sectional imaging are among the features associated with increased nerve-injury risk. These findings should guide the choice between conventional extraction, modified techniques such as coronectomy where appropriate, referral, and explicit informed consent [39].

**Figure 3:**
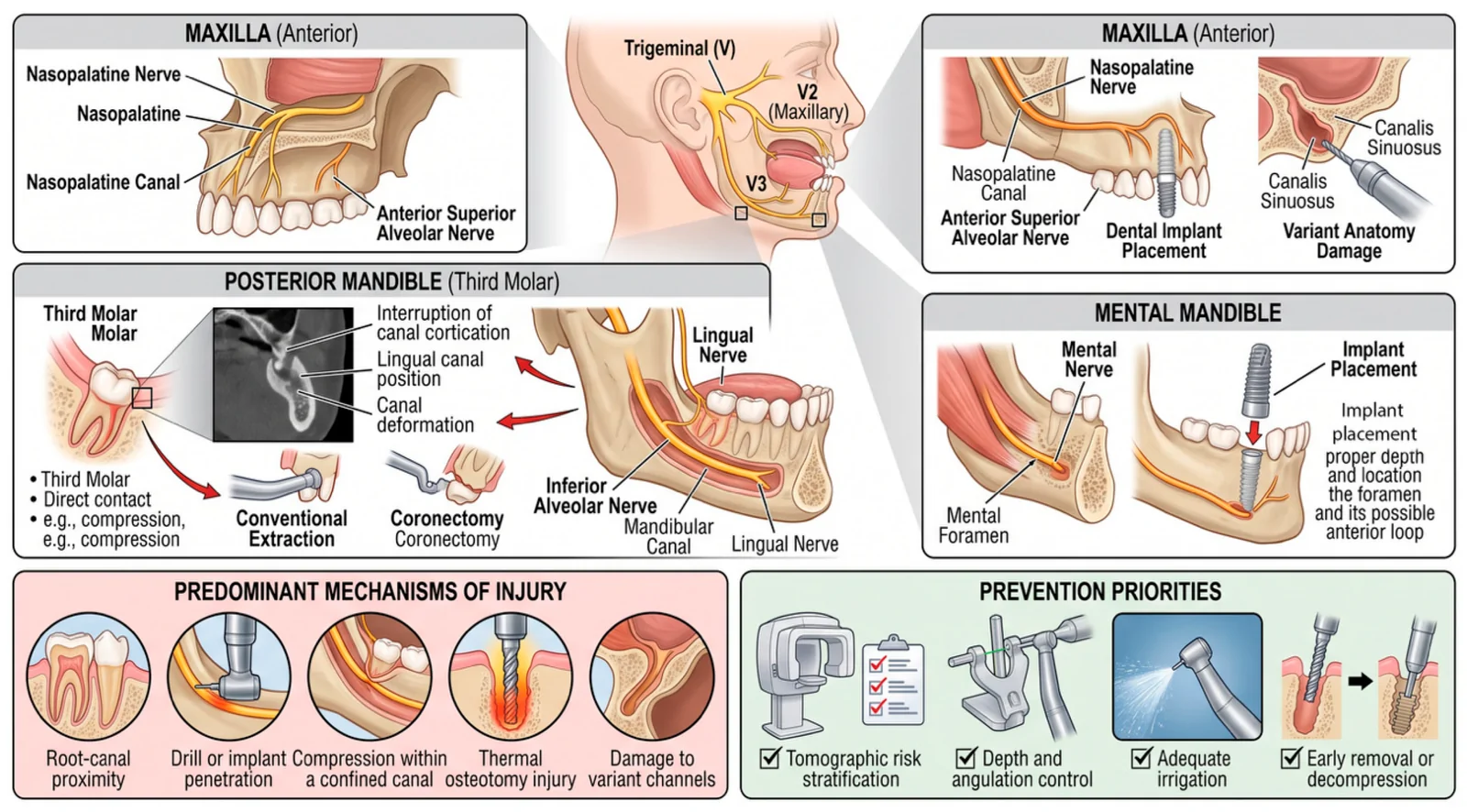
Procedure-specific cranial and peripheral nerves at risk in craniofacial and skull base surgery. Abbreviations: CN, cranial nerve; ICA, internal carotid artery.

The principal procedure-specific injury profiles are summarized in Table 2. Although the underlying mechanisms are shared, the nerves at greatest risk and the resulting deficits differ substantially according to the surgical corridor and procedure, as illustrated in Figure 3.

## 6. Preventive Strategies

### 6.1. Preoperative Planning and Risk Stratification

Prevention begins with a nerve-specific surgical plan. Imaging should define the relationship between the lesion, foramina, canals, vascular structures, and expected operative corridor. In lateral skull base tumors, the surgeon should anticipate displacement of the facial and cochlear nerves and plan the sequence of internal debulking, capsule dissection, and stimulation. In endoscopic surgery, thin or eroded bone over the carotid artery, optic canal, and cavernous sinus nerves should be identified before drilling [1,6]. When conventional magnetic resonance imaging (MRI) does not clearly separate a distorted cranial nerve from tumor, probabilistic tractography and newer trajectory-distribution methods may help anticipate nerve position; however, false-positive trajectories, limited spatial resolution, and intraoperative anatomical shift remain important constraints [40,41,42,43].

In mandibular surgery, cross-sectional imaging is used to assess the course of the mandibular canal, cortical integrity, the relationship of pathology or osteotomy lines to the nerve, and the feasibility of nerve preservation. If oncological resection will require inferior alveolar nerve sacrifice, reconstructive planning should include anticipated gap length, availability of proximal and distal stumps, graft diameter, and coordination with bony reconstruction [27]. For dentoalveolar procedures, cone-beam computed tomography can identify high-risk canal-root relationships and other tomographic features that should influence consent and operative planning [37,39].

Preoperative facial grading and selective electrophysiological testing can establish a functional baseline when tumor-related neuropathy or subsequent deterioration is a concern. Aimoni et al. demonstrated the value of comparing clinical facial function with electroneurography before and after parotid surgery [44]. Wiertel-Krawczuk et al. similarly combined clinical assessment with electroneurography, EMG, and blink-reflex testing to characterize branch-specific dysfunction and recovery [45]. These studies principally inform perioperative assessment and prognosis; they do not provide controlled evidence that intraoperative monitoring itself prevents injury.

### 6.2. Operative Technique

Core protective measures include meticulous exposure, early anatomical identification, avoidance of blind traction, and preservation of the vascular envelope of the nerve. Tumor debulking should precede forceful capsule mobilization. Dissection should proceed from known anatomy toward distorted anatomy, and instruments should be directed away from the expected nerve trajectory. Near neural structures, drilling should be intermittent and continuously irrigated, and coagulation should be minimized [1,6]. In craniofacial surgery, comparable principles include direct protection of exposed sensory branches and selection of drilling systems that minimize soft-tissue entanglement and thermal transfer [24,28].

During sagittal split osteotomy, complete osteotomy, controlled splitting, gentle nerve mobilization, and fixation that does not compress or penetrate the canal are central. When the nerve is visualized in the proximal segment, deliberate release should be performed only when necessary and without excessive manipulation. Any recognized laceration should be documented and referred for immediate or early microsurgical management [17,29]. These measures align with broader orthognathic recommendations concerning controlled exposure, osteotomy completion, segment handling, and fixation placement [20,21,32].

### 6.3. Intraoperative Neuromonitoring and Mapping

Intraoperative neuromonitoring should be planned as a sequence of baseline acquisition, nerve localization, surveillance during dissection, response to alerts, and end-of-procedure verification. Modality selection must follow the structures at risk. Free-running electromyography (EMG) detects motor-unit activity associated with manipulation, whereas triggered EMG maps accessible motor nerves and tests evoked responses. Facial motor evoked potentials may provide complementary information about the corticobulbar pathway. When hearing preservation is attempted, auditory monitoring, including brainstem auditory evoked responses, addresses a pathway that facial EMG cannot assess [8,9,11]. Continuous EMG recording should not be confused with continuous stimulation, and the absence of spontaneous EMG activity does not establish preserved conduction [12].

The 2018 Congress of Neurological Surgeons guideline recommends facial nerve monitoring during vestibular schwannoma surgery and supports eighth nerve monitoring when hearing preservation is attempted [9]. These recommendations should not be interpreted as equivalent evidence for every craniofacial procedure. Before critical dissection, the team should confirm electrode placement, reproducible responses, stimulation location and intensity, and the effects of neuromuscular blockade. Initial and final responses should be obtained under comparable conditions and documented by muscle or branch where feasible [11,12,46]. For an extracranial motor nerve, distal stimulation may remain positive despite injury to a more proximal segment; it therefore cannot alone establish integrity across the entire operative field [12].

A sustained deterioration or loss of a previously reproducible response should trigger an immediate pause in the potentially injurious maneuver and joint assessment by the surgeon, neurophysiologist, and anesthesiologist. The response should include release of traction or compression, review of recent coagulation or drilling, irrigation when thermal injury is suspected, and correction of relevant perfusion or physiological disturbances. In parallel, electrode connections, probe contact, current delivery, stimulus artifact, neuromuscular blockade, and the position of stimulation relative to the suspected lesion should be checked. Repeated mapping and proximal stimulation, when safely accessible, help distinguish a technical failure from a localized conduction disturbance. Persistent changes should prompt reassessment of the dissection plane and the balance between further resection and functional risk, rather than automatic nerve sacrifice [9,11,12,46].

Parotid surgery provides a useful example of protocol standardization, although its extracranial setting differs from intracranial skull base dissection. In a retrospective series of 55 patients, Duque et al. proposed a sequence of multichannel recording, initial main-trunk and branch stimulation, repeat testing after dissection, and troubleshooting of suspected signal loss [46]. The study supports the feasibility of a reproducible workflow, but its uncontrolled design and incomplete recordings do not establish a reduction in postoperative palsy or a validated universal alarm threshold. Its principles of comparable baseline and final testing are transferable; exact stimulation settings and stopping rules should not be imported uncritically into other procedures.

The operative record should connect each substantial signal change with the maneuver being performed, the technical and physiological checks undertaken, the corrective action, and any subsequent recovery. An end-of-procedure assessment should document both anatomical continuity and the conditions under which final responses were obtained. Standardized reporting is also necessary before automated detection systems or multichannel datasets can be judged clinically useful; a well-characterized dataset is not, by itself, evidence that an algorithm improves patient outcomes [8,11,12,23,46].

**Figure 4:**
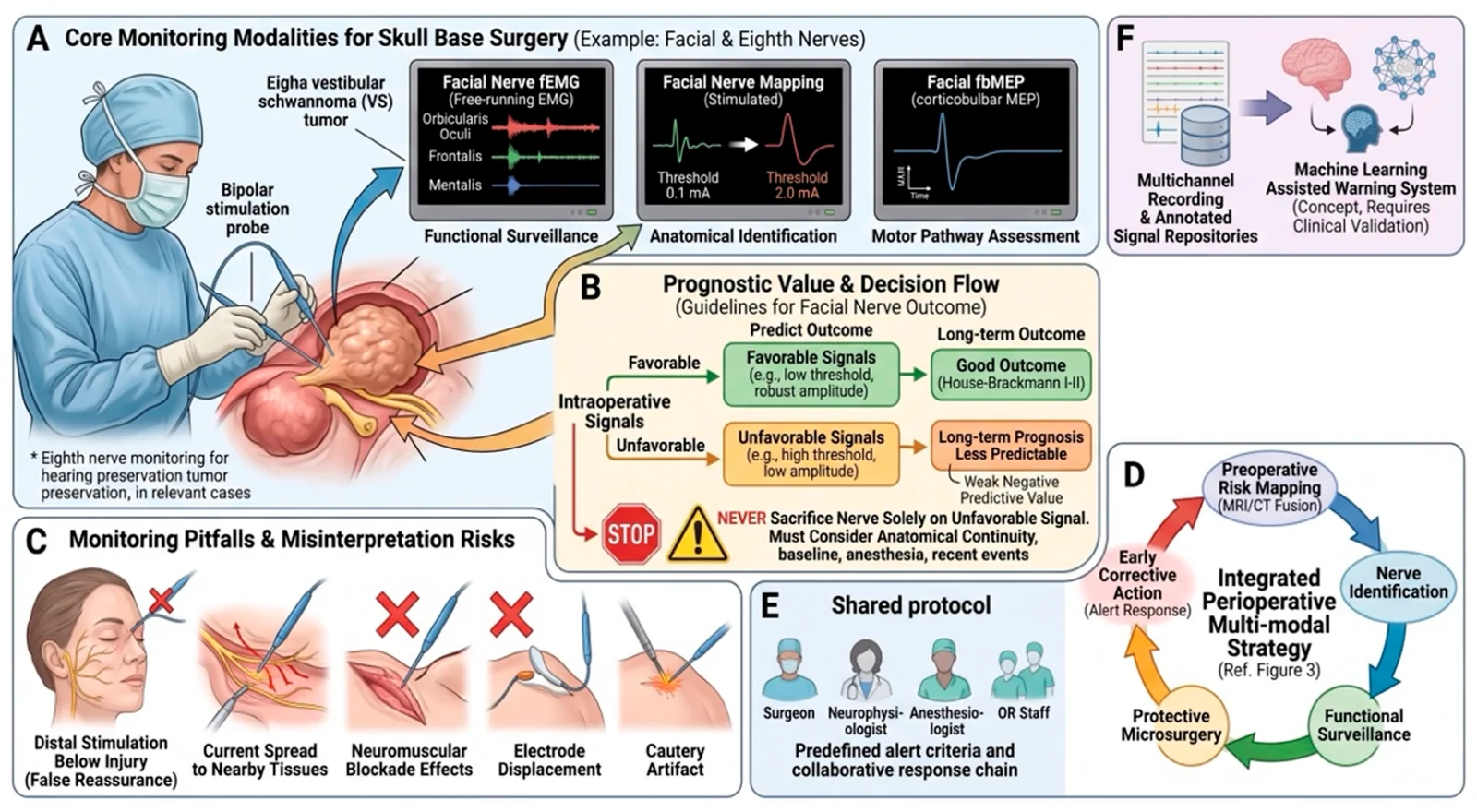
**Multimodal strategy for the prevention of iatrogenic nerve injury during skull-base surgery.** (**A**) Core intraoperative monitoring modalities, illustrated for facial and vestibulocochlear nerve preservation, including free-running facial electromyography, stimulation-based facial nerve mapping, and facial corticobulbar motor-evoked potentials. (**B**) Interpretation of intraoperative signals and their prognostic implications for facial nerve outcomes. Unfavorable signals alone should not justify nerve sacrifice; interpretation must incorporate anatomical continuity, baseline function, anesthetic conditions, and recent surgical events. (**C**) Common monitoring pitfalls and sources of misinterpretation, including stimulation distal to the injury site, current spread to adjacent tissues, neuromuscular blockade, electrode displacement, and electrocautery artifacts. (**D**) Integrated perioperative strategy combining preoperative risk mapping, intraoperative nerve identification, functional surveillance, protective microsurgical techniques, and early corrective action in response to alerts. (**E**) Shared multidisciplinary protocol defining alert criteria and coordinated responses among the surgeon, neurophysiologist, anesthesiologist, and operating-room staff. (**F**) Multichannel recording and annotated signal repositories supporting the development of machine-learning-assisted warning systems, which require clinical validation. Abbreviations: CT, computed tomography; EMG, electromyography; MEP, motor-evoked potential; MRI, magnetic resonance imaging; OR, operating room.

These preventive measures should be applied as a continuous multimodal perioperative strategy integrating risk mapping, nerve identification, functional surveillance, protective microsurgery, and early corrective action (Figure 4).

## 7. Postoperative Evaluation and Clinical Decision-Making

Postoperative assessment should establish a documented baseline as early as clinically feasible. Facial function should be graded using a reproducible instrument such as the House-Brackmann scale, Sunnybrook system, or eFACE, supplemented by photographs or standardized video when possible [10,26,47]. Examination must include eye closure, corneal protection, resting tone, oral competence, smile excursion, synkinesis, and patient-reported functional and psychosocial impact.

Where postoperative facial dysfunction persists or its severity is uncertain, electrophysiological testing should be selected and timed according to the clinical question, such as axonal loss, residual motor-unit activity, or reinnervation. Serial electroneurography and EMG complement clinical grading but should not be interpreted without reference to the interval from injury and the operative findings. The parotid studies by Aimoni et al. and Wiertel-Krawczuk et al. illustrate the added information provided by longitudinal testing and branch-specific assessment, rather than reliance on a single global facial score [44,45].

Peripheral trigeminal injuries require mapping of the affected sensory territory and standardized neurosensory testing. Light touch, pinprick, temperature discrimination, brush direction, and two-point discrimination can be integrated into the Medical Research Council sensory scale. Serial testing is more informative than a single examination because the trajectory of recovery helps distinguish neurapraxia from more severe injury [16,17,29,35,36]. Pain quality, spontaneous versus evoked symptoms, and functional interference should be recorded separately from sensory threshold. The examination should also account for procedure-specific territories, including lingual, infraorbital, mental, and optic-related symptoms after orthognathic surgery, as well as any pre-existing orthodontic deficit [19,22,34].

Immediate exploration is indicated when transection is observed, a nerve has been unintentionally sacrificed, or imaging and operative findings strongly suggest entrapment or severe compression that can be corrected. For a nerve that remains in continuity, a period of observation is reasonable if function is stable or improving. Failure of meaningful recovery, worsening dysesthesia, or evidence of neuroma should prompt referral to a peripheral nerve or facial reanimation specialist. No single timing threshold applies to every injury, but prolonged delay reduces the quality of recipient stumps and, for motor nerves, risks irreversible motor end-plate degeneration [10,29,47].

**Figure 5:**
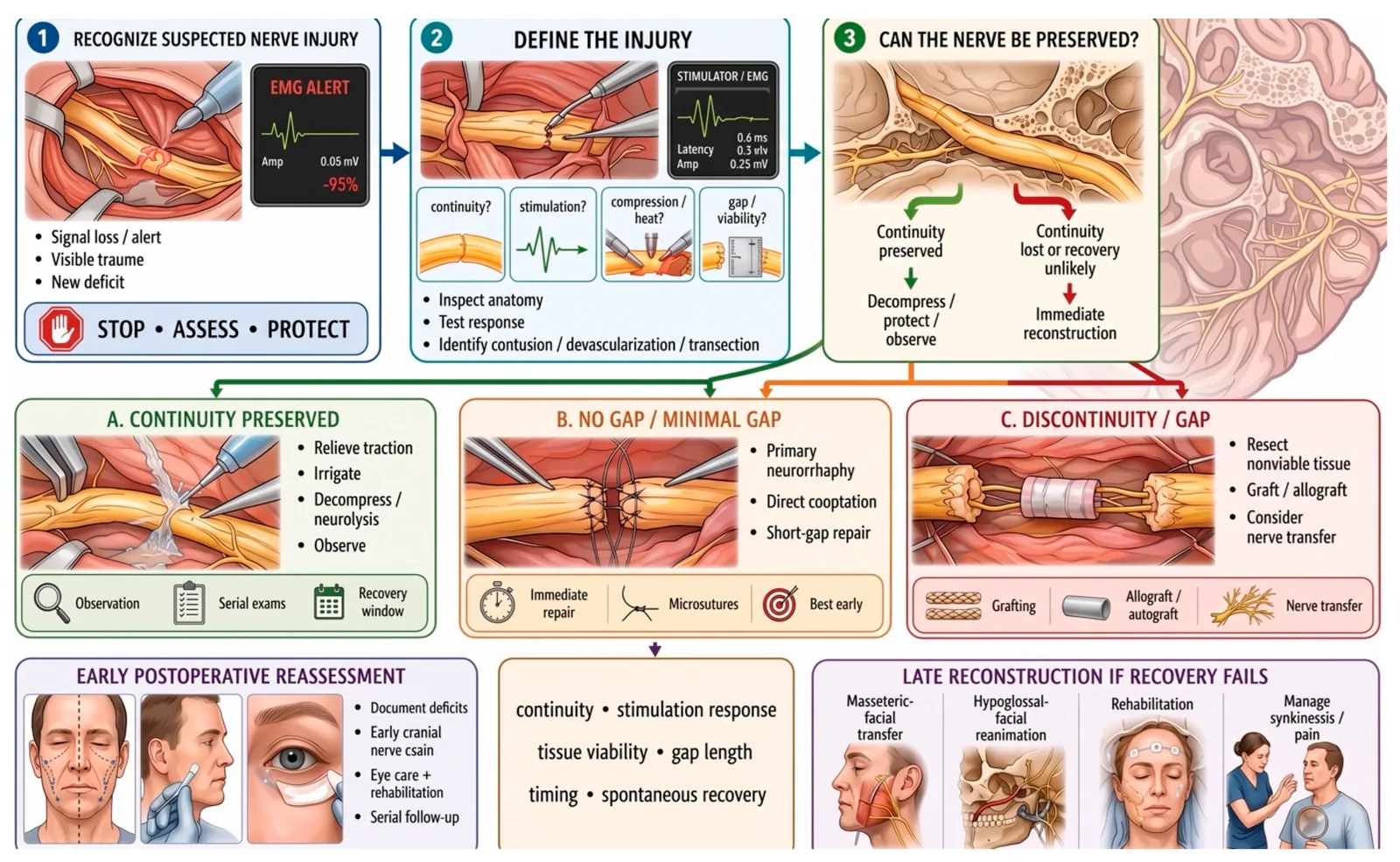
Intraoperative decision-making after suspected nerve injury. Abbreviation: EMG, electromyography.

Once nerve injury is suspected, management should follow a structured decision pathway based on nerve continuity, physiological responsiveness, tissue viability, and the feasibility of immediate repair (Figure 5).

## 8. Repair, Reanimation, and Rehabilitation

**Figure 6:**
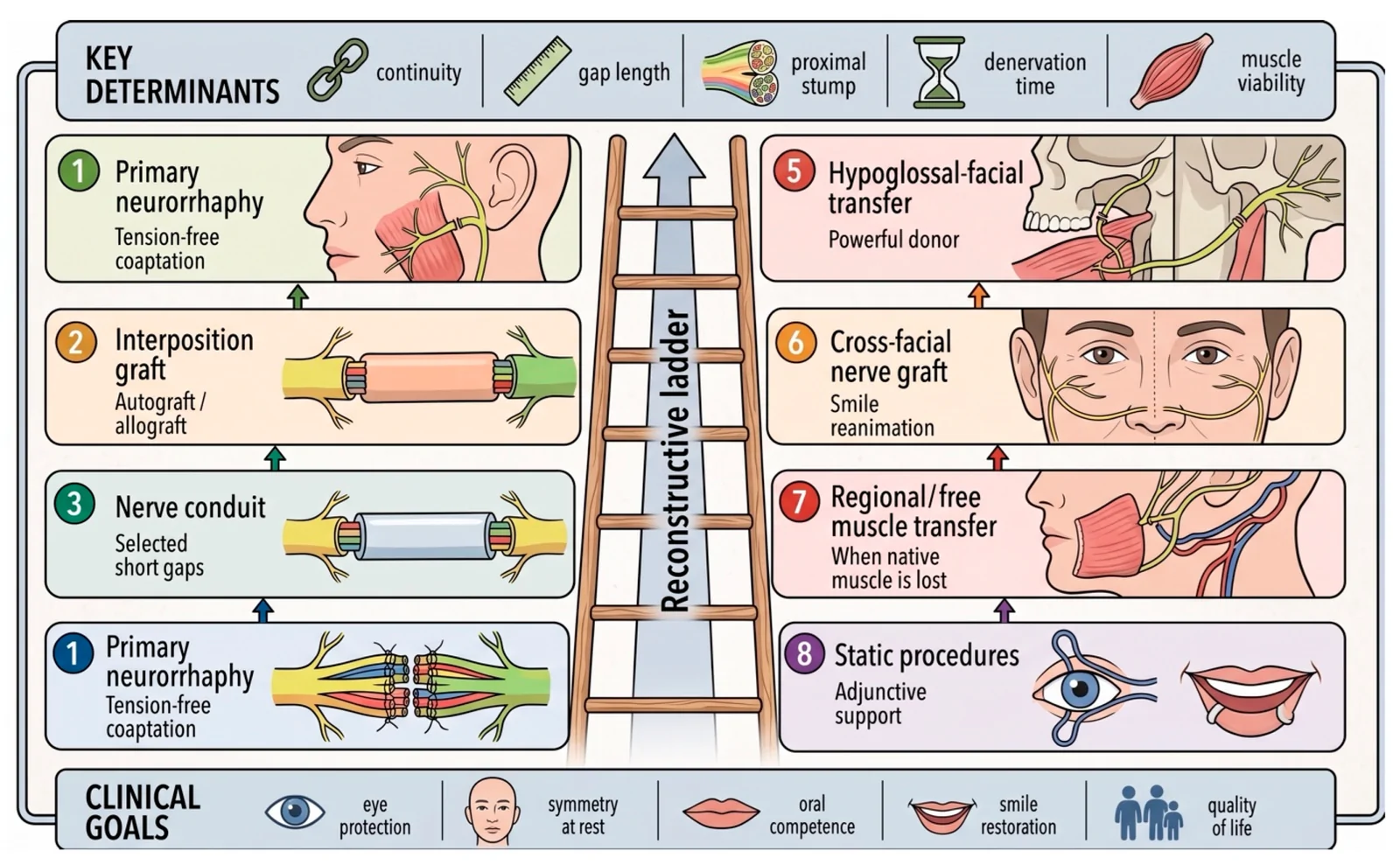
Reconstructive ladder for cranial and peripheral facial nerve injuries.

The available reconstructive options can be conceptualized as a ladder determined by nerve continuity, gap length, donor availability, denervation duration, and target-muscle viability (Figure 6).

### 8.1. Direct Repair and Interposition Grafting

The fundamental reconstructive principles are accurate debridement to healthy fascicles, tension-free coaptation, appropriate alignment, minimal suture burden, and a vascularized tissue bed. Direct end-to-end neurorrhaphy is preferred when the ends can be approximated without tension. When a gap remains, autologous cable grafts or processed nerve allografts may be used according to nerve type, diameter, defect length, and the surgical setting [10,27,29]. Regenerative and bioengineering research after cerebellopontine angle surgery supports continued investigation of conduits, cellular therapies, neurotrophic strategies, and biomaterial-assisted reconstruction, but clinical evidence remains less mature than that for established microsurgical repair [7].

Immediate reconstruction is particularly advantageous when a nerve is sacrificed during tumor resection because the injury level is known, the stumps are available, and fibrosis has not yet developed. In mandibular resection, immediate long-span inferior alveolar nerve allografting produced functional sensory recovery in 80% of reconstructed patients at one year and in 94% of patients with benign pathology, compared with 7% in a non-reconstructed group [27]. Although the retrospective design and pathology differences require caution, the magnitude of the difference supports planned reconstruction whenever oncological and anatomical conditions permit.

### 8.2. Facial Nerve Transfer and Dynamic Reanimation

When the proximal facial nerve stump is unavailable or unsuitable, donor nerve transfer is used to reinnervate viable facial musculature. The masseteric nerve offers a strong, nearby motor source and often produces rapid smile excursion, whereas hypoglossal-facial transfer provides powerful tone but may carry donor-site morbidity involving tongue movement, speech, or swallowing. Partial and end-to-side hypoglossal techniques were developed to reduce this morbidity [10,47,48].

A large retrospective series of 153 patients after lateral skull base or middle ear surgery compared masseteric-facial and hypoglossal-facial anastomosis. Masseteric transfer was associated with earlier recovery and better function at six months, while later outcomes were similar between groups. In both groups, an interval of six months or less between palsy and reanimation was associated with better results [47]. A systematic review of hypoglossal-facial techniques similarly found generally favorable recovery but highlighted heterogeneity in technique, timing, and outcome assessment [48].

Composite or zone-based reconstruction may combine a masseteric input for dynamic midface movement, partial hypoglossal input for tone, a cross-facial graft for spontaneous movement, and static procedures for ocular and oral support. A small cohort using immediate multi-donor reanimation demonstrated improved symmetry and movement, illustrating the potential of patient-specific reconstruction but not yet providing comparative evidence [26].

### 8.3. Peripheral Trigeminal Nerve Repair

For inferior alveolar and lingual nerve injuries, treatment is determined by the mechanism, clinical trajectory, pain phenotype, gap length, and operative findings. Neurolysis may be sufficient for a nerve in continuity that is externally scarred or compressed. Direct neurorrhaphy is appropriate for a short, tension-free defect; longer gaps require autograft or allograft reconstruction [16,29,35,36]. Implant-related injuries require early recognition of mechanical compression or direct canal violation, because prompt removal or decompression may be time-sensitive even when later microsurgical repair remains possible [37].

Systematic reviews report useful sensory recovery after repair in selected patients, but heterogeneous definitions of recovery, surgical techniques, and predominantly retrospective designs preclude a single generalizable success rate [16,29]. A recent meta-analysis of permanent lingual nerve injury included 1358 injuries and reported a pooled functional sensory recovery rate of 92% after intervention [35]. An earlier focused meta-analysis found that repair within six months was associated with a higher probability of recovery, whereas conduit use did not independently improve outcomes [36]. Differences in eligibility criteria and timing definitions explain some apparent inconsistency between reviews and reinforce the need for individualized interpretation.

In patients with persistent deficits after sagittal split osteotomy, microsurgical exploration revealed discontinuity, partial severance, neuroma-in-continuity, and compression injuries. After repair, 50% achieved complete recovery and 35% useful sensory recovery at a minimum of one year [17]. These findings support referral when recovery is absent, or symptoms remain unacceptable, rather than assuming that all postoperative paresthesia will resolve spontaneously.

### 8.4. Ocular Protection, Rehabilitation, and Adjunctive Care

Rehabilitation should begin with protection of the affected organ and an assessment of residual function, rather than waiting for the completion of reconstruction. In acute facial paralysis, incomplete eye closure requires lubrication and appropriate mechanical protection, with ophthalmological assessment and escalation to temporary tarsorrhaphy or eyelid loading when exposure persists. Ocular protection remains necessary during reinnervation and after reconstructive procedures; a stronger smile does not necessarily indicate adequate blink or corneal protection. Static suspension may improve resting symmetry and oral competence while neural recovery develops [10].

Facial rehabilitation should be individualized to the stage of denervation or reinnervation and to the reconstructive procedure. Early care emphasizes education, relaxation, soft-tissue mobility, and protection; when voluntary activity returns, specialist-guided retraining focuses on small, selective movements rather than forceful, undifferentiated contraction. Mirror feedback and, when available, surface EMG biofeedback help patients recognize intended activation and unwanted co-contraction. After donor nerve transfer, training is adapted to the donor motor command and progresses toward functional facial movement [10,49,50]. The updated review by Khan et al. supports facial exercise therapy but includes heterogeneous etiologies, protocols, and outcome measures; much of the evidence concerns Bell’s palsy, so it should not be treated as direct proof of efficacy for every postsurgical lesion or transfer [50].

When synkinesis develops, the goal shifts from generating stronger movement to improving selective activation and suppressing involuntary co-contraction. International recommendations place facial training, preferably incorporating biofeedback, as first-line treatment; targeted botulinum toxin can be added when troublesome hyperactivity limits function or retraining. Injection sites and doses should be individualized, with attention to unintended weakness and impairment of eye closure or oral competence. Selective neurectomy or myectomy is reserved for carefully selected refractory cases. These recommendations are clinically useful but rely largely on observational evidence and expert consensus rather than robust head-to-head trials [49].

For patients with lower cranial nerve dysfunction, rehabilitation must also address swallowing safety, voice, and nutritional intake. Laryngological and swallowing assessment should guide therapy rather than assuming that dysphonia and dysphagia share a single correctable mechanism. Guidelines for unilateral vocal fold paralysis support early voice therapy in suitable patients and consideration of medialization at diagnosis when aspiration risk is present. However, restoring glottic closure may not resolve dysphagia caused by multiple cranial nerve injuries or impaired pharyngeal sensation; these patients require broader multidisciplinary assessment and individualized swallowing support [51].

Sensory rehabilitation after peripheral trigeminal injury combines protection of numb oral tissues, education to avoid thermal or biting injury, graded desensitization for hypersensitivity, and sensory re-education as function returns. Follow-up should document the sensory territory, stimulus detection and discrimination, pain, chewing, speech, and accidental soft-tissue injury. Persistent neuropathic pain warrants individualized treatment and specialist input rather than reliance on sensory thresholds alone. Evidence for specific rehabilitation regimens after trigeminal repair is less developed than evidence for surgical reconstruction; these measures support function and safety but should not be presented as established accelerators of axonal regeneration [2,16,29,35,36].

Progress should be assessed using both clinician-rated and patient-reported measures. Standardized photographs or video, Sunnybrook or eFACE grading, and a synkinesis-specific measure such as the Synkinesis Assessment Questionnaire can track outcomes not captured by gross motor recovery alone. Goals should include eye comfort and protection, oral competence, intelligible speech, eating, social participation, and the patient’s priorities. A therapist-supervised home program, review of adherence, and access to psychological support are integral to longitudinal care, while the frequency and duration of therapy should be individualized because an optimal regimen has not been established [10,49,50].

**Figure 7:**
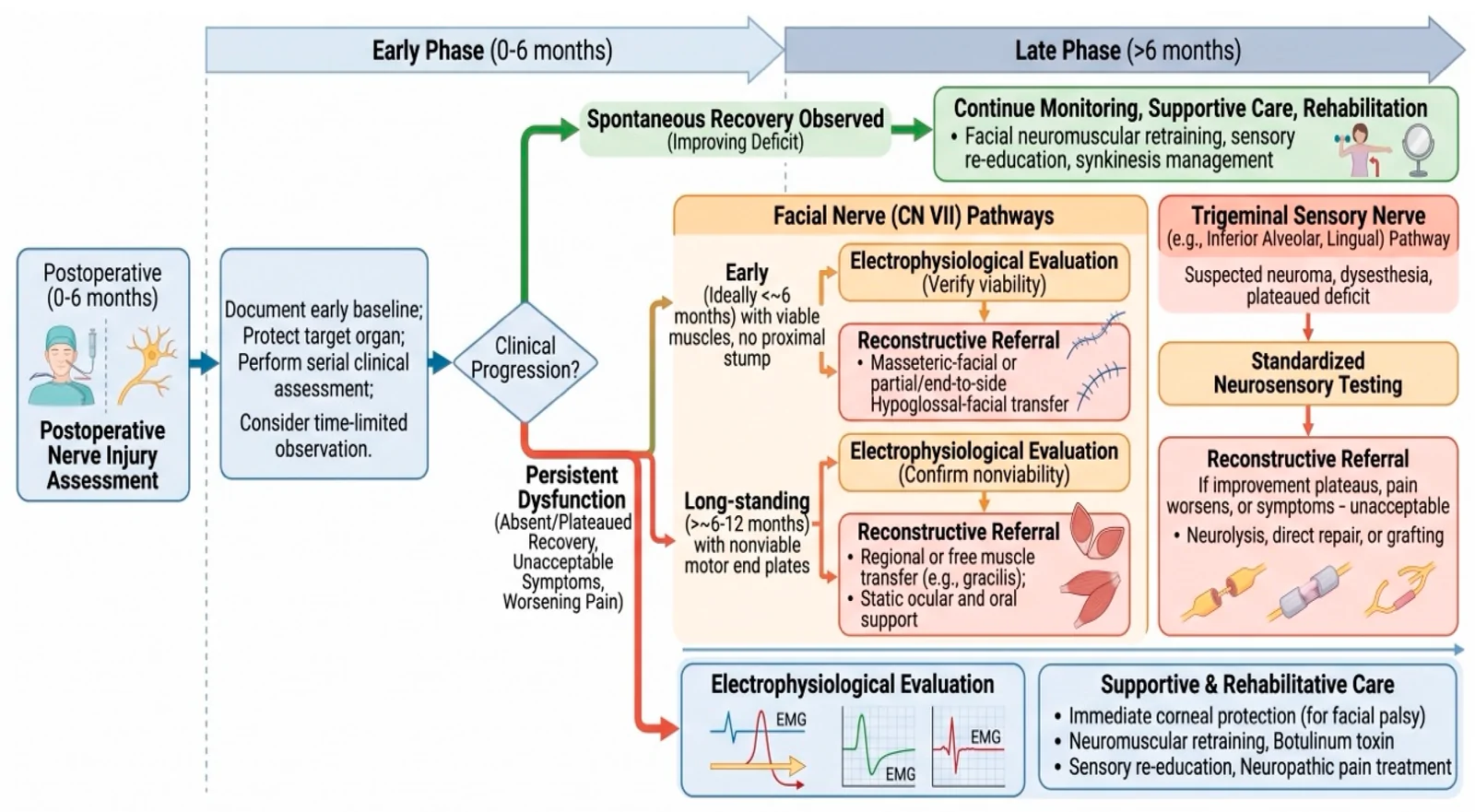
Timeline of postoperative evaluation and functional recovery after nerve injury. Abbreviation: EMG, electromyography.

A practical management framework based on structural continuity, target-organ viability, and clinical trajectory is presented in Table 3. Furthermore, a structured follow-up timeline helps distinguish expected spontaneous recovery from persistent dysfunction requiring electrophysiological evaluation, reconstructive referral, or long-term rehabilitation (Figure 7).

## 9. Discussion

The practical contribution of this review is to connect recurring injury mechanisms with the decisions they require across specialties, rather than to propose a novel classification. A facial nerve stretched during vestibular schwannoma dissection and an inferior alveolar nerve compressed during mandibular osteotomy differ anatomically, but both require assessment of mechanical stress, perfusion, continuity, and the trajectory of function. The resulting pathway is clinically specific: recognize and reverse potentially reversible insults, distinguish technical monitoring failure from neural deterioration, reconstruct confirmed discontinuity when feasible, and link serial assessment to timely specialist referral [1,9,10,12,16,29]. For practical use, Table 1, Table 2 and Table 3 and Figure 4, Figure 5, Figure 6 and Figure 7 bring these domains together, linking the operative injury mechanism to prevention, postoperative assessment, and individualized reconstruction and rehabilitation.

Neuromonitoring is a central component of prevention only when its findings change operative behavior. Its value lies in combining nerve localization with repeated functional assessment and a shared response to deterioration, not simply in documenting that monitoring equipment was used. The evidence and recommendations are strongest and most directly applicable within defined procedures, such as facial nerve monitoring during vestibular schwannoma surgery [8,9]. A favorable end-of-procedure response may support prognosis, but an unfavorable response does not establish irreversible failure; conversely, distal stimulation or current spread can produce false reassurance [9,11,12]. Thus, anatomical continuity, baseline function, stimulation site, recent surgical events, and signal reproducibility must be interpreted together.

The parotid literature helps distinguish three separate questions: whether electrophysiology detects dysfunction, whether a monitoring workflow is feasible, and whether its use improves outcomes. Aimoni et al. and Wiertel-Krawczuk et al. primarily inform the first question through preoperative and postoperative assessment [44,45]. Duque et al. address the second by proposing standardized intraoperative recordings and a response to suspected signal loss [46]. None of these studies alone establishes the third question through a controlled comparison. Accordingly, their findings support reproducible assessment and communication without justifying claims that monitoring eliminates facial palsy or that a single alarm criterion applies across operative corridors.

The clinically relevant monitoring endpoint is therefore an actionable warning interpreted under reliable recording conditions. Free-running EMG, triggered responses, and pathway-specific evoked potentials provide different information and should not be treated as interchangeable tests. When deterioration occurs, the response should link technical verification to release of traction, reassessment of the operative plane, and consideration of the functional consequences of continued resection. Evidence from supratentorial eloquent-tumor surgery provides indirect support for integrated mapping and monitoring, but its effect estimates cannot be extrapolated to cranial nerve preservation or used to validate a skull base monitoring protocol [8,9,11,12,52].

The evidence also supports a strong distinction between recognized discontinuity and an injury-in-continuity. Observed transection or planned sacrifice should generally be reconstructed immediately when oncological safety and local anatomy permit. Immediate repair avoids stump retraction, fibrosis, and prolonged denervation and is supported by both facial reanimation principles and the marked sensory recovery reported after immediate inferior alveolar nerve allografting [10,27]. In contrast, an anatomically intact nerve with partial dysfunction should not be sacrificed or transferred solely because of an unfavorable intraoperative signal. Serial clinical and electrophysiological assessment is more appropriate unless the nerve is known to be structurally nonviable [9].

Timing is repeatedly associated with outcome, but the literature does not define one universal deadline. Facial muscle viability makes delay especially consequential for motor reconstruction. The Lauda cohort showed better results when reanimation was performed within six months, and contemporary management reviews similarly emphasize early restoration of neural input [10,47]. For peripheral trigeminal injuries, some reviews favor intervention after approximately three months without improvement, while newer analyses suggest that useful recovery may still occur after later repair and that timing interacts with injury type, preoperative function, and surgical technique [16,29,35,36]. The practical implication is not that every deficit requires immediate surgery, but that observation should be active, time-limited, and supported by standardized serial testing.

Outcome measurement remains a major weakness. Facial nerve studies use House-Brackmann, Sunnybrook, eFACE, photographic analysis, or surgeon-defined categories, while trigeminal studies vary in their use of Medical Research Council grades, two-point discrimination, pain scales, and patient satisfaction. This heterogeneity limits comparison and may obscure clinically important outcomes such as spontaneous smile, synkinesis, corneal protection, altered taste, speech difficulty, and neuropathic pain. Rehabilitation should therefore be evaluated alongside reconstruction rather than treated as an unspecified co-intervention. Future studies should combine standardized clinical testing with validated patient-reported measures, document treatment exposure and adherence, and report functional outcomes at consistent follow-up intervals [16,29,49,50].

The evidence base has additional limitations. Most reconstructive studies are retrospective and frequently single-center, making them vulnerable to selection bias, historical controls, surgeon-specific expertise, and incomplete adjustment for injury severity. Randomized trials are uncommon and may be ethically or practically difficult when dealing with recognized transection or planned nerve sacrifice. Systematic reviews therefore pool heterogeneous case series and cannot fully resolve the relative benefits of competing graft materials, donor nerves, or timing strategies [11,16,29,35,36,48]. Reported recovery rates show that reconstruction can benefit selected patients, not that techniques are equivalent. The present narrative selection process also introduces selection and language bias; it is not an exhaustive systematic review, and no formal study-level risk-of-bias assessment is presented. The absence of screening-stage counts limits reconstruction of the historical selection process. Accordingly, complication frequencies and recovery rates cited from individual reports should be interpreted within their original procedural, patient, and follow-up contexts, not as pooled benchmarks for expected risk or treatment success across craniofacial and skull base surgery.

Future neuromonitoring studies should specify the operation and nerve segment, baseline clinical and electrophysiological function, recording configuration, anesthetic conditions, stimulation site and intensity, alert definition, and the action taken after each alert. They should distinguish technical or physiological artifacts from persistent neural changes and relate both to early and long-term functional outcomes. Prospective registries should additionally record continuity, injury mechanism, reconstruction, rehabilitation exposure, and patient-reported disability, allowing monitoring performance and clinical benefit to be evaluated separately. Comparative reconstructive studies need consistent recovery definitions and adjustment for gap length, denervation duration, and target-muscle viability. Cranial nerve tractography research should quantify localization error and the impact of intraoperative anatomical shift rather than relying only on apparent tract visualization [8,11,12,23,40,41,42,43,46,49,50].

## 10. Conclusions

This review supports a mechanism- and continuity-based approach to nerve injury in craniofacial and skull base surgery. A concerning monitoring change should prompt a pause, technical and physiological checks, and reversal of potentially injurious traction, compression, or thermal exposure before further dissection. Reproducible baseline and final recordings, interpreted in relation to the stimulation site and operative events, are more informative than an isolated threshold or waveform. Recommendations remain procedure-specific; parotid monitoring protocols and perioperative electrophysiological studies complement, but do not substitute for, skull base evidence.

The pivotal treatment distinction is confirmed discontinuity versus an anatomically preserved nerve with impaired function. Recognized transection or necessary nerve sacrifice favors immediate tension-free reconstruction when oncological and anatomical conditions permit. Poor electrical responsiveness alone does not justify sacrificing an intact nerve. Instead, serial clinical and appropriately timed electrophysiological assessment should guide observation, referral, and reconstruction while target muscles remain viable.

Rehabilitation is part of this decision pathway, with immediate ocular or swallowing protection where indicated, stage-specific facial or sensory retraining, treatment of synkinesis and pain, and measurement of patient-perceived function. The available evidence does not establish a universal alert threshold, a single deadline for reconstruction, or superiority of one rehabilitation regimen across all procedures. The most useful next studies will test whether defined monitoring responses and coordinated follow-up improve nerve-specific function, rather than merely reporting device use or anatomical preservation.

## Figures and Tables

**Table 1:** Shared mechanisms of nerve injury and practical preventive responses.

Mechanism	Representative Operative Contexts	Typical Injury Pattern or Consequence	Prevention and Immediate Response
Traction and compression	Cerebellopontine angle or lateral skull base tumor dissection; sagittal split osteotomy; retraction around foramina and canals.	Neurapraxia, axonotmesis, delayed palsy, sensory loss, or dysesthesia; dysfunction may occur despite preserved continuity.	Debulk before mobilizing the capsule; avoid blind or sustained traction; release compression; pause after monitoring changes; reassess with proximal stimulation [1,8,11,17,20,21].
Devascularization and ischemia	Skeletonization or coagulation near the facial nerve vasa nervorum; cavernous or paraclival dissection; manipulation within tight bony canals.	Delayed or immediate dysfunction without visible transection; conduction deterioration may be progressive.	Preserve the vascular envelope; minimize coagulation and circumferential dissection; use intermittent manipulation; maintain systemic perfusion and correlate signal changes with surgical events [1,6,8,12].
Thermal and energy-related injury	High-speed drilling, ultrasonic instruments, bipolar or monopolar electrosurgery, and implant osteotomy preparation.	Demyelination, axonal injury, vascular compromise, or abrupt loss of conduction.	Use continuous cold irrigation and intermittent drilling; favor controlled diamond-burr work near nerves; avoid direct contact and lateral thermal spread [1,6,24,25].
Direct mechanical injury and planned nerve sacrifice	Osteotomy, screw or drill penetration, sharp dissection, displaced bone, implant violation, or soft-tissue entanglement.	Contusion, fascicular disruption, partial laceration, entrapment, or complete transection.	Map the expected trajectory; protect exposed branches; direct instruments away from the nerve; inspect and document suspected injury; repair recognized transection immediately when feasible [2,17,24,27,28,29].

**Table 2:** Procedure-specific nerve injury profiles and prevention priorities.

Surgical Setting	Principal Nerves at Risk	Predominant Mechanisms	Typical Deficits and Prevention Priorities
Lateral skull base and cerebellopontine angle surgery	Facial (VII) and vestibulocochlear (VIII) nerves; trigeminal and lower cranial nerves in selected lesions.	Tumor-related distortion or adherence, traction, devascularization, thermal injury, drilling, or planned sacrifice.	Facial weakness or paralysis, hearing loss, corneal exposure, facial sensory symptoms, or dysphagia. Priorities: internal debulking before capsule mobilization, early nerve mapping, vascular preservation, and multimodal monitoring [1,8,9,10,11,12,15].
Endoscopic endonasal skull base surgery	Optic (II), oculomotor (III), trochlear (IV), trigeminal divisions (V1-V2), abducens (VI), vidian, and lower cranial nerves depending on the approach.	Drilling around thin or dehiscent bone, cavernous sinus or paraclival dissection, vascular injury, direct manipulation, or CSF-related traction.	Visual loss, diplopia, facial sensory disturbance, dry eye or nasal dysfunction, and lower cranial nerve deficits. Priorities: define bony variants and vascular relationships, expose from known anatomy, and use triggered mapping for motor nerves in distorted fields [3,6,25,30,31].
Orthognathic and mandibular surgery	Inferior alveolar, lingual, long buccal, and mental nerves; less commonly infraorbital, optic, or motor cranial nerves in unfavorable fractures.	Retraction, osteotomy and splitting, segment displacement, fixation, compression, or an unintended fracture trajectory.	Lower lip, chin, or tongue sensory loss; dysesthesia; altered taste; speech or mastication difficulty. Priorities: cross-sectional imaging, complete and controlled osteotomy, gentle segment handling, canal-safe fixation, and serial neurosensory testing [17,18,19,20,21,32,33,34].
Dentoalveolar and implant surgery	Inferior alveolar, lingual, mental, nasopalatine, and anterior superior alveolar nerves.	Root-canal proximity, drill or implant penetration, compression within a canal, thermal osteotomy injury, or entrapment.	Numbness, dysesthesia, neuropathic pain, or altered taste. Priorities: tomographic risk stratification, depth and angulation control, adequate irrigation, and early removal or decompression when mechanical violation is suspected [22,24,29,35,36,37,38,39].

Abbreviations: CSF, cerebrospinal fluid; V1, ophthalmic division of the trigeminal nerve; V2, maxillary division of the trigeminal nerve.

**Table 3:** Practical management framework for postoperative cranial and peripheral trigeminal nerve injury.

Clinical Scenario	Recommended Management and Timing	Evidence and Key Considerations
Anatomically intact nerve with a stable or improving deficit	Document an early baseline; protect the target organ; perform serial clinical and, when appropriate, electrophysiological assessment. Use active, time-limited observation rather than immediate reanimation.	An unfavorable end-of-case EMG response has limited negative predictive value and should not alone justify nerve sacrifice or early transfer [9,12].
Observed transection with approximable ends	At the index operation, debride to healthy fascicles and perform tension-free end-to-end neurorrhaphy with minimal suture burden in a vascularized bed.	Immediate repair avoids stump retraction, fibrosis, and an additional denervation interval [10,29].
Segmental defect or planned nerve sacrifice	Perform immediate interposition autografting or processed allografting when the nerve type, diameter, gap length, and local bed are suitable; coordinate with tumor and bony reconstruction.	Immediate long-span inferior alveolar nerve allografting achieved functional sensory recovery in 80% overall and 94% after benign-disease resection, versus 7% without reconstruction [27].
Complete facial paralysis with viable mimetic muscles and no usable proximal facial stump	Use masseteric-facial or partial/end-to-side hypoglossal-facial transfer, ideally within approximately 6 months; consider cross-facial or zone-based combinations when spontaneous movement is a priority.	Masseteric transfer produced faster early recovery, whereas later outcomes seem similar to hypoglossal transfer. Selection must balance excursion, resting tone, spontaneity, and donor morbidity [10,26,47,48].
Long-standing facial paralysis with nonviable motor end plates	Use regional or free functional muscle transfer with static ocular and oral support; individualize the reconstructive sequence and rehabilitation.	The viability of recipient musculature determines whether nerve-based reanimation remains feasible [10].
Persistent inferior alveolar or lingual nerve deficit, dysesthesia, or suspected neuroma	Use standardized serial neurosensory testing initially; refer when improvement plateaus, pain worsens, or symptoms remain unacceptable. Select neurolysis, direct repair, or grafting according to operative findings and gap length.	Useful sensory recovery is reported in selected patients, with heterogeneous outcome definitions; a recent meta-analysis reported 92% recovery after intervention for permanent lingual nerve injury. Timing remains individualized [16,17,29,35,36].
Supportive and rehabilitative care	Institute immediate corneal protection and assess swallowing safety when indicated. Tailor facial motor or sensory retraining to recovery stage; use biofeedback and selected botulinum toxin treatment for synkinesis, with individualized pain management and psychosocial support.	Combine clinical grading with patient-reported function, synkinesis, and psychosocial outcomes. Rehabilitation evidence is heterogeneous and not uniformly specific to postsurgical injury [2,10,26,35,49,50,51].

Abbreviations: EMG, electromyography.

## Data Availability

No new data were created or analyzed in this study. All data discussed are available in the cited published literature.

## Abbreviations

The following abbreviations are used in this manuscript:

CN	cranial nerve
CSF	cerebrospinal fluid
CT	computed tomography
EMG	electromyography
ICA	internal carotid artery
MEP	motor evoked potential
MRI	magnetic resonance imaging
V1	ophthalmic division of the trigeminal nerve
V2	maxillary division of the trigeminal nerve
